# Supplementary material for: Causal Relationship between Aging and Anorexia Nervosa: A White-Matter-Microstructure-Mediated Mendelian Randomization Analysis
Source: Biomedicines. 2024 Aug 16;12(8):1874. doi: 10.3390/biomedicines12081874 (PMC11351342; doi:10.3390/biomedicines12081874)
Supplement: Supplementary file 1 [file biomedicines-12-01874-s001.zip › Supplementary Figure/Figure S4/Singe SNP plot of MR analysis for TL to ubm-b-1893.pdf]

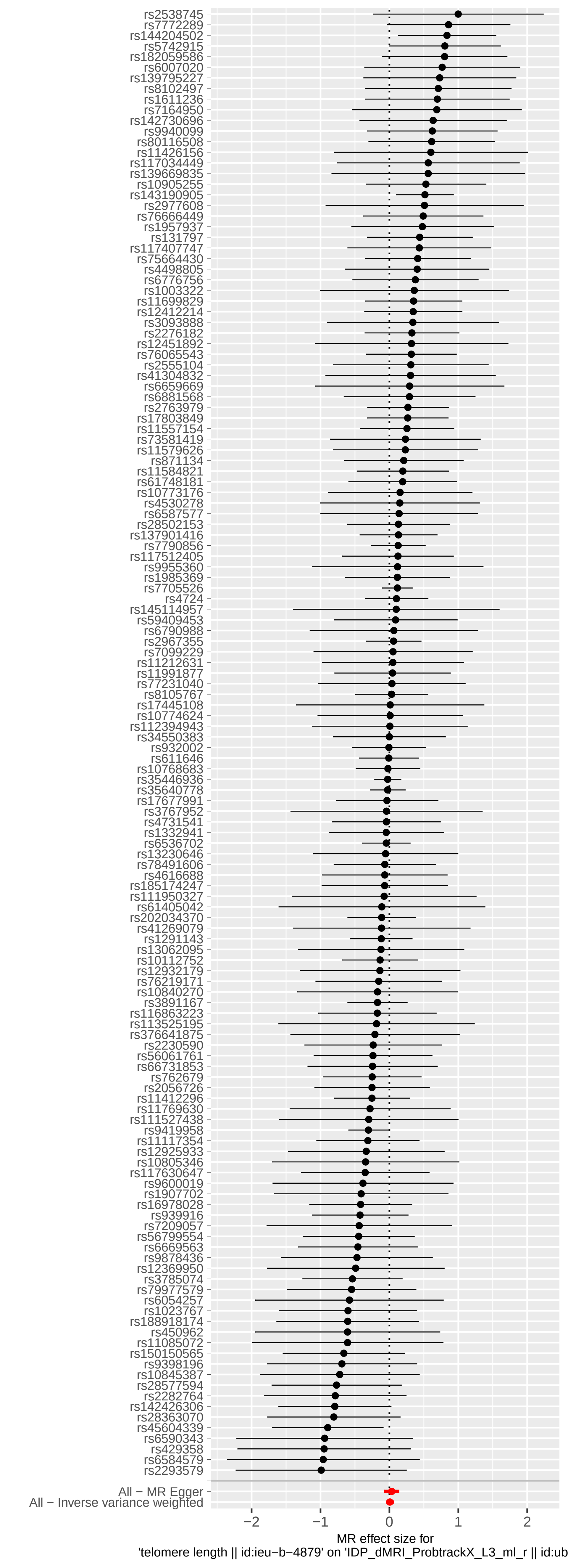
rs2538745
rs7772289
rs144204502
rs5742915
rs182059586
rs6007020
rs139795227
rs8102497
rs1611236
rs7164950
rs142730696
rs9940099
rs80116508
rs11426156
rs117034449
rs139669835
rs10905255
rs143190905
rs2977608
rs76666449
rs1957937
rs131797
rs117407747
rs75664430
rs4498805
rs6776756
rs1003322
rs11699829
rs12412214
rs3093888
rs2276182
rs12451892
rs76065543
rs2555104
rs41304832
rs6659669
rs6881568
rs2763979
rs17803849
rs11557154
rs73581419
rs11579626
rs871134
rs11584821
rs61748181
rs10773176
rs4530278
rs6587577
rs28502153
rs137901416
rs7790856
rs117512405
rs9955360
rs1985369
rs7705526
rs4724
rs145114957
rs59409453
rs6790988
rs2967355
rs7099229
rs11212631
rs11991877
rs77231040
rs8105767
rs17445108
rs10774624
rs112394943
rs34550383
rs932002
rs611646
rs10768683
rs35446936
rs35640778
rs17677991
rs3767952
rs4731541
rs1332941
rs6536702
rs13230646
rs78491606
rs4616688
rs185174247
rs111950327
rs61405042
rs202034370
rs41269079
rs1291143
rs13062095
rs10112752
rs12932179
rs76219171
rs10840270
rs3891167
rs116863223
rs113525195
rs376641875
rs2230590
rs56061761
rs66731853
rs762679
rs2056726
rs11412296
rs11769630
rs111527438
rs9419958
rs11117354
rs12925933
rs10805346
rs117630647
rs9600019
rs1907702
rs16978028
rs939916
rs7209057
rs56799554
rs6669563
rs9878436
rs12369950
rs3785074
rs79977579
rs6054257
rs1023767
rs188918174
rs450962
rs11085072
rs150150565
rs9398196
rs10845387
rs28577594
rs2282764
rs142426306
rs28363070
rs45604339
rs6590343
rs429358
rs6584579
rs2293579
All − MR Egger
All − Inverse variance weighted
−2
−1
0
1
2
MR effect size for
'telomere length || id:ieu−b−4879' on 'IDP_dMRI_ProbtrackX_L3_ml_r || id:ub
